# A heart rate variability-driven framework for depression screening leveraging emotion-elicited autonomic divergence

**DOI:** 10.1186/s40101-025-00414-6

**Published:** 2025-12-24

**Authors:** Zhibin Zhu, Xuanyi Wang, Yifei Xu, Wanlin Chen, Jing Zheng, Shulin Chen, Hang Chen

**Affiliations:** 1 College of Biomedical Engineering & Instrument Science, Zhejiang University, Hangzhou, China; 2 Department of Psychology and Behaviorial Sciences, Zhejiang University, Hangzhou, China; 3 School of Medicine, Hangzhou City University, Hangzhou, China; 4 Nanhu Brain-Computer Interface Institute, Hangzhou, China; 5 Zhejiang Provincial Key Laboratory of Cardio-Cerebral Vascular Detection Technology and Medicinal Effectiveness Appraisal, Hangzhou, China

**Keywords:** Depression, Heart rate variability (HRV), Sadness, Emotional induction

## Abstract

**Objective:**

Depression manifests significant emotional dysregulation, characterized by heightened sadness susceptibility and attenuated happiness responsiveness in individuals with depression (IWD). This study employs structured emotion induction protocols to analyze physiological response disparities between IWD and healthy controls (HC) across multiple affective states, establishing empirical foundations for optimizing affective computing-based depression screening.

**Methods:**

Dual-phase statistical identification was conducted using Mann–Whitney *U* tests: initially verifying emotion elicitation validity by comparing HRV features between emotional states and resting conditions, subsequently detecting IWD/HC response differences within each emotion. Machine learning frameworks were then constructed leveraging HRV features and intergroup differential response patterns.

**Results:**

Comparative analysis revealed generally consistent directional patterns and response magnitudes across groups for most features, while critical divergences emerged characterized by heightened sadness reactivity in IWD alongside attenuated happiness responsiveness. Implemented models achieved 76.8% accuracy (AUC = 0.772, 95% CI 0.699–0.841) under sadness-specific conditions, outperforming anger/happiness-induced models (≈ 70% accuracy) and substantially surpassing resting-state baselines.

**Conclusion:**

Systematic investigation of HRV-mediated elicitation patterns through discrete emotion induction confirms clinically significant differential responsiveness between groups, empirically validating heightened sadness susceptibility in IWDs.

**Significance:**

These findings offer valuable guidance for refining affective computing-based depression screening algorithms, while contributing to the mechanistic understanding of disorder-specific physiological responses to emotional stimuli.

**Supplementary Information:**

The online version contains supplementary material available at 10.1186/s40101-025-00414-6.

## Highlights

1. Multi-state emotion induction differences: using HRV feature analysis to quantify the distinct autonomic response patterns of individuals with depression (IWD) and healthy controls (HC) across four emotional states (sadness/happiness/fear/anger) and resting conditions.
2. Validated sadness hyper-reactivity: compared to HCs, IWDs exhibited stronger physiological reactivity to sadness stimuli and weaker reactivity to happiness, validating findings from other non-physiological studies.
3. Superior sadness-evoked identification: an affective computing framework was developed, achieving 76.8% depression screening accuracy under sadness induction.
4. Dual theoretical and clinical advancement: providing novel insights and actionable optimization pathways for next-generation depression screening.

**Supplementary Information:**

The online version contains supplementary material available at 10.1186/s40101-025-00414-6.

## Introduction

Depression is a common mental disorder [1]. Individuals with depression (IWDs) often experience a decline in quality of life [2], reduced work efficiency, cognitive dysfunction [3], and even intense suicidal ideation [4]. Globally, the age-standardized prevalence rate of depression is 3440 cases per 100,000 people, making it the second most prevalent mental disorder after anxiety disorders [5]. This situation has been exacerbated by the outbreak of COVID-19 and the global economic downturn [6]. Therefore, depression-screening methods are crucial research topics in psychology.

Depression is a mental disorder that is highly associated with emotions. IWDs exhibit significant differences in emotion induction compared to healthy controls (HCs). For instance, Mizrahi [7] found that, compared with HCs, IWDs are more likely to feel sad and amplify their feelings of sadness when listening to emotional music clips and reporting their own experiences. Then, they are also less likely to experience joy. Similarly, Arens [8] used musical materials and self-reports to demonstrate that IWDs exhibited a higher propensity for sadness. These emotion-induced differences have been identified by some researchers, which has led to the gradual development of depression-screening methods based on affective computing. Compared with the most commonly used depression scales, such as the Hamilton Depression Rating Scale [9] (HAMD) and the Patient Health Questionnaire-9 [10] (PHQ-9), and the methods based on resting-state physiological signals [11–14], the depression-screening methods based on the combination of affective computing and physiological signals can be specifically designed to optimize the signal acquisition process. This allows for more accurate and effective screening of depression.

In the current researches, Özmen [15] obtained fMRI images of participants' brains by having them view positive and negative emotion-inducing images inside an MR scanner. Using weighted-3D-DWT (Discrete Wavelet Transform) and machine learning methods for depression classification, an accuracy rate of up to 97.3% was achieved. Lyu [16] conducted a study in which participants were sequentially exposed to six stimuli tasks containing specific emotional content in the form of images, videos, or text. Galvanic skin response (GSR) signals were collected, and a machine learning model based on Support Vector Machine (SVM) was constructed to differentiate between patients with bipolar depression (BPD), major depressive disorder (MDD), and healthy individuals. The model achieved an accuracy of 78% in distinguishing MDD from healthy individuals and 59% in the three-class classification. Lan [17] utilized electroencephalogram (EEG) signals for this purpose. Nine movie clips containing happy, sad, and neutral emotions were used as stimuli. It was found that the average activation levels of the alpha, beta, and gamma frequency bands in the frontal and temporal lobes of IWDs differed from those of healthy individuals. Based on these findings, a convolutional neural network model was constructed, which demonstrated a sensitivity of 81.93% and a specificity of 91.69% for detecting happy emotions.

In addition to depression-screening methods based on complex physiological signals such as fMRI and EEG, methods that involve more convenient signal acquisition are gradually receiving increasing attention, such as those based on Heart Rate Variability (HRV) features derived from electrocardiogram (ECG) signals. Zhang [18] proposed a classification algorithm based on neuro-fuzzy methods, utilizing several HRV features to differentiate between IWDs and HCs. Through the Multimodal Affective Content (MAC) test, which includes emotional and other types of stimuli, continuous ECG signals were collected for 800 s, and HRV features were extracted. The study ultimately found that the SDNN (Standard Deviation of Normal-to-Normal intervals) and VLF (very low frequency) features exhibited significant differences between IWDs and HCs. Similarly, Kim [19] employed the MAC test to acquire ECG data and extract HRV features. The study discovered that the most interesting video and meditation accompanied by natural sounds within the MAC test were effective for classifying IWDs and HCs. Utilizing a neuro-fuzzy algorithm, an average accuracy rate of 86.6% was achieved.

In summary, methods for depression screening based on affective computing have progressively garnered significant research interest. Concurrently, standardized protocols for methods such as MAC have been developed, thereby facilitating the translation of the aforementioned research into practical applications. However, it was observed that not all stimuli in protocols like MAC effectively distinguish between HCs and IWDs, as high redundancy persists. For instance, only the most interesting video and the meditation were effective in Kim’s study [19]. Consequently, this study aims to expand upon the work of Mizrahi and Arens by conducting a feature-level analysis of HRV to compare responses of HCs and IWDs to diverse emotional stimuli. A further objective is to explore a novel approach for depression screening, potentially leading to an optimized protocol derived from existing MAC frameworks.

## Methods

The key steps of the research methods in this study mainly include (1) collecting scale information and ECG signals under multiple emotional states from patients with depression and healthy control subjects; (2) performing denoising and other preprocessing operations on the collected ECG signals, and extracting 9 commonly used features of HRV; (3) utilizing the Mann–Whitney *U* test and distribution plots, examining the emotional elicitation profiles within both IWDs and HCs, specifically investigating differences in the magnitude and potentially the directionality of elicited responses between the two groups; (4) finally, based on machine learning algorithms, investigating whether it is possible to construct a depression screening method based on emotional induction differences and HRV.

### Emotional stimulation materials and subjects

This study has been approved by the Medical Ethics Committee of the Department of Psychology and Behavioral Sciences at Zhejiang University (Zhejiang University Psychological Ethics Review [2022] No. 059). The experiment is based on Ekman’s discrete emotion theory [20] and refers to authoritative emotion databases such as the DECAF database established by Abadi's research team [21]. Several emotional video clips were selected to induce calmness, happiness, sadness, fear, and anger. Our research team recruited approximately 70 participants to evaluate the emotion-inducing effects of the candidate video clips, and ultimately determined the video materials that could significantly induce emotions. The effectiveness of these emotion-inducing materials for emotion induction experiments was fully validated [22]. The specific material selection and the emotions they induce are shown in Table 1.

**Table 1:** Emotional stimulation materials

Source movie	Duration (s)	Emotion	Scene description
/	192	Calmness	Daily life of a family
Gandhi	123	Anger	Indian attorney gets thrown out of a first-class train compartment
My Bodyguard	101	Anger	Group of thugs provoke a teenager
Up	67	Happiness	Carl—a shy, quiet boy—meets the energetic Ellie
The Truman Show	60	Happiness	Truman and his lover go to the beach for a romantic evening
Wall-E	93	Happiness	all-E and Eve spend a romantic night together
The Shining	78	Fear	A child enters hotel room searching for his mom
Black Swan	62	Fear	A woman notices paranormal activity around her
My Girl	66	Sadness	A young girl cries at her friend’s funeral
Bambi	166	Sadness	The fawn Bambi’s mother is killed by a deer hunter

In the formal experimental phase, this study recruited undergraduate or graduate students from Zhejiang University to participate in the experiment. All participants were required to have normal vision, hearing, and perceptual abilities. Before the experiment, participants were asked to accurately and seriously complete the Patient Health Questionnaire-9 (PHQ-9) scale. The PHQ-9 is a self-administered tool developed for primary care settings, aligning with the DSM-IV (Diagnostic and Statistical Manual of Mental Disorders, Fifth Edition) diagnostic criteria for major depression [23]. It has been widely established as a concise and effective instrument for detecting both the presence and severity of depressive disorders, making it highly valuable for initial screening and exploratory research. In standard use, a PHQ-9 total score ≤ 4 is interpreted as indicative of a non-depressed (healthy) population, whereas a score ≥ 10 signals a high probability of depressiom. Consequently, participants in the present study were designated as HCs (healthy controls) in the former case and as IWDs (individuals with suspected depression) in the latter. Based on the content that needs to be studied in this article, a priori power analysis was conducted with G*Power 3.1.9.7 [24] for a mixed-design ANOVA (between-factor: HCs vs IWDs; within-factor: five emotions). According to the default parameter settings(α = 0.05, power = 0.95, effect size *f* = 0.25, and correlation among repeated measures = 0.5), G*Power indicated a minimum requirement of 32 participants (i.e., ≥ 16 per group). Allowing for modest oversampling and contingent on volunteer availability, recruitment was terminated once 30 valid IWD cases had been secured. The final sample comprised 157 valid participants: 30 IWDs and 66 HCs. Detailed information of the participants is shown in Table 2.

**Table 2:** Subjects’ information

Variable	Value
Number of all subjects	157
Number of subjects (IWDs/HCs)	30/66
Age (IWDs / HCs)	22.96 ± 2.43/21.80 ± 2.61
Gender (IWDs Male/IWDs Female/HCs Male/HCs Female)	12/18/27/39
Education level	Undergraduate or graduate students

### Experimental process and data acquisition

**Fig. 1:**
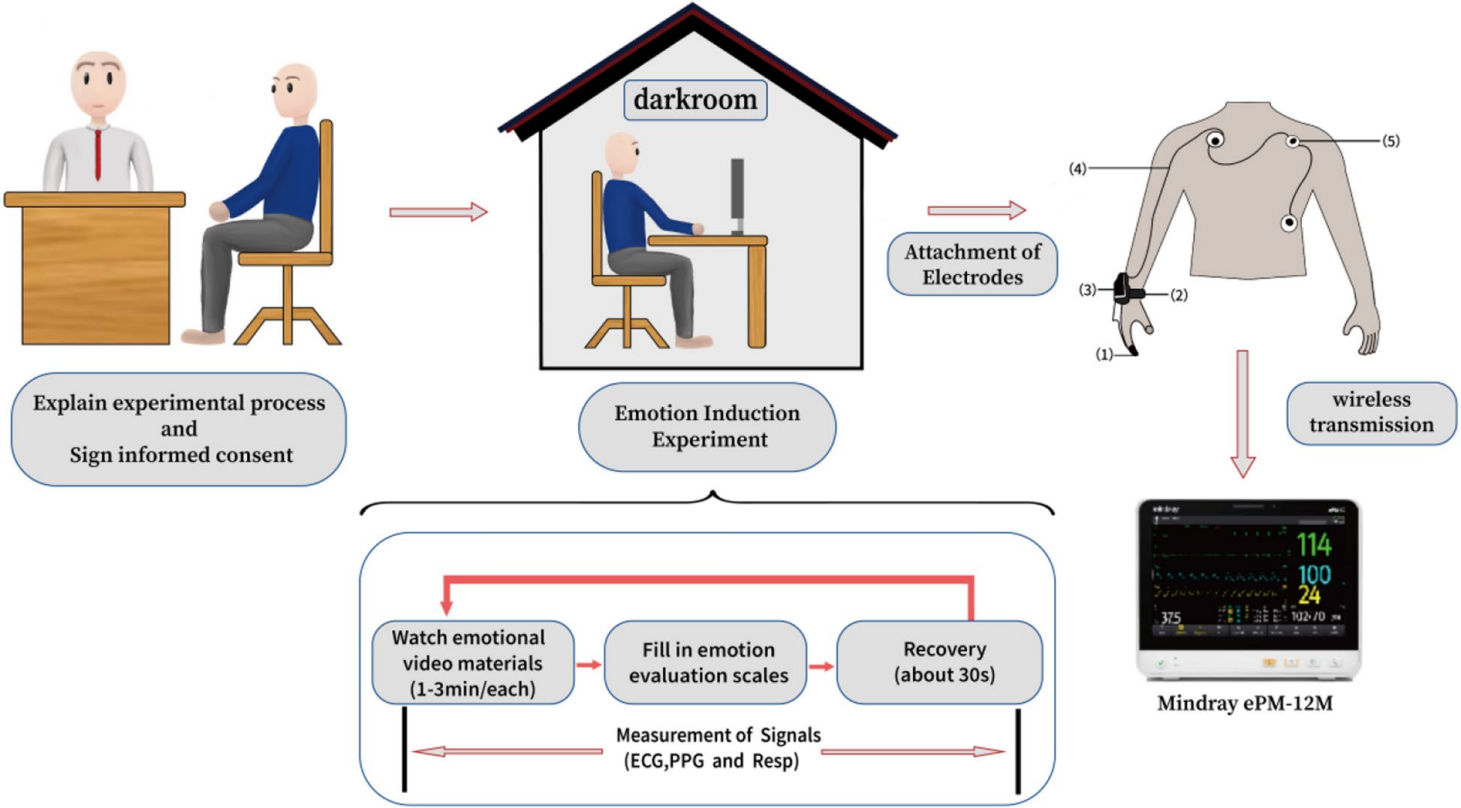
Experimental process

The experimental procedure is shown in Fig. 1. Before the experiment, participants were thoroughly introduced to the experimental procedures and precautions, asked to sign an informed consent form, and required to accurately and seriously complete the PHQ-9 scale. Subsequently, the participants were escorted to a darkroom (an isolated room capable of being blacked out), only equipped with a desk, a chair, a computer, and a display. Inside, ECG electrodes were placed on each participant with the experimenter’s assistance. The three electrodes were positioned at the following sites: inferior to the midpoint of the left clavicle, inferior to the midpoint of the right clavicle, and inferior to the midpoint of the lowest rib on the lower left abdomen. A Bluetooth transmission module was affixed to the participant’s left wrist. The ECG signal, sampled at 500 Hz, was monitored in real-time on a physiological signal monitor (ePM-12M, Mindray, China) located outside the darkroom. Signal quality was verified visually on the monitor; this was ensured by adjusting the participant’s posture and making minor electrode adjustments as necessary. Following this, the lights of the darkroom were turned off, leaving only the light from the display. The experimenter then exited the room, and the participant, maintaining a comfortable seated position, proceeded to independently complete the experiment as per the pre-experiment instructions.

The experimental procedure required participants to complete a sequence of seven video trials, each following a fixed protocol: video viewing, self-assessment, and a rest period. The videos were presented in a predetermined sequence of emotional valences: calm, positive, negative, positive, negative, positive, and negative. While the sequence of valences was fixed, the specific video exemplars for each category were randomized for each participant. Here, “positive” denotes videos eliciting happiness, whereas “negative” encompasses videos designed to evoke sadness, fear, or anger. This design ensured that every participant was exposed to all five target emotional states: calmness, happiness, sadness, fear, and anger. After each video, participants’ subjective responses were assessed using the Self-Assessment Manikin (SAM). A set of six 9-point scales was administered for each video, comprising four scales for rating the intensity of the discrete emotions (happiness, sadness, fear, anger), one scale for arousal, and one for valence.

### Signal processing and feature extraction

Given that the ECG signals were acquired using medical-grade equipment, the subsequent preprocessing was straightforward. The processing pipeline consisted of the following steps: first, a band-pass filter (0.5–100 Hz) and a 50-Hz notch filter were applied to remove baseline wander and powerline interference. Subsequently, R-peaks were precisely identified using the algorithm implemented in the Neurokit2 [25] The continuous signal was then segmented according to the timestamps corresponding to the presentation of each experimental stimulus. Finally, for each stimulus segment, non-overlapping, 1-min epochs were extracted from the end of the segment for subsequent analysis. Considering the length limitations of each epoch, some HRV features could not be calculated or were deemed invalid [26, 27]. Therefore, this study focused on several common computable HRV features, and the respiratory rate used to determine HRV respiratory effects, as shown in Table 3.

**Table 3:** Several typical HRV features

Number	Features	Description
1	MeanNN	Mean value of NN intervals
2	SDNN	Standard deviation of NN intervals
3	RMSSD	Root mean square of successive differences
4	CVNN	Standard deviation of NN intervals (SDNN) divided by mean value of NN intervals (MeanNN)
5	CVSD	Root mean square of successive differences (RMSSD) divided by mean of NN intervals (MeanNN)
6	LFn	The normalized low frequency, obtained by dividing the low frequency power by the total power
7	HFn	The normalized high frequency, obtained by dividing the low frequency power by the total power
8	LF/HF	The ratio obtained by dividing the low frequency power by the high frequency power
9	Resp_Rate	Respiratory rate indirectly calculated through HRV-HF

This research investigates the magnitude and directionality of emotional elicitation by employing a baseline-referenced standardization approach. The calm state serves as the individual-specific baseline to quantify elicited emotional responses. To mitigate substantial inter-individual variability and enhance the discernibility of emotional responses, we applied a z-score-based standardization referenced to the calm state. This method is mathematically defined as follows:

Let *s* denote a participant, and let ${{F}}_{{{s}}, {{calm}}}=\{{{F}}_{{{s}}, {{calm}}}^{({1})}, \, {{F}}_{{{s}}, {{calm}}}^{({2})}, ...,{{F}}_{{{s}}, {{calm}}}^{({{n}})}\}$ represent the set of feature values for participant *s* during the calm state, where *n* ≥ 1 indicates the whole segment of calmness having multiple epochs. The within-subject mean *μ*_*s,calm*_ and standard deviation *σ*_*s,calm*_ of the Calm state are computed as

1 $${\mu }_{{{s}}, \, {{calm}}}=\frac{1}{{{n}}}{\sum }_{{{i}}={1}}^{{n}}{{{F}}}_{{{s}}, \, {{calm}} \, }^{({{i}})}$$

2 $${\sigma }_{{{s}}, {{calm}}}=\sqrt{\frac{1}{{{n}}-{1}}{\sum }_{{{i}}={1}}^{{n}}{({{F}}_{{{s}}{, }{{calm}}}^{({{i}})}-{\mu }_{{{s}}, {{calm}}})}^{2}}$$

Subsequently, for any feature value *F*_*s,epoch*_ observed in the same participant *s* during any epoch mentioned above (e.g., happiness or sadness), the standardized feature ${{F}}_{{{s}},{{epoch}}}^{{std}}$ is derived using:

3 $${{F}}_{{{s}}, \, {{epoch}}}^{{std}}=\frac{{{F}}_{{{s}}, {{epoch}}}-{\mu }_{{{s}}, {{calm}}}}{{\sigma }_{{{s}}, \, {{calm}}}}$$

This approach ensures that the standardization is performed at the individual level, using participant-specific Calm state parameters. As a result, ${{F}}_{{{s}},\ {{epoch}}}^{{std}}$ represents the deviation of the emotional feature from the participant’s calm baseline, expressed in units of the calm state’s standard deviation.

### Methodology

This study focuses on investigating emotional elicitation in both IWDs and HCs, along with differences in elicitation efficacy between the groups. Consequently, the initial analytical step involves examining HRV feature responses across both IWDs and HCs under various emotional states, assessed through feature distribution analysis and statistical comparison. The Mann–Whitney *U* test, implemented using the SciPy package in Python [28], was employed at this stage to evaluate the statistical significance of observed differences in HRV features attributable to emotional elicitation or underlying inter-group distinctions. Subsequently, the *p*-values obtained from the Mann–Whitney *U* tests were subjected to false discovery rate (FDR) correction to account for multiple comparisons. To strengthen the statistical conclusions, effect sizes were calculated using Cohen's d. A result was deemed statistically significant and practically meaningful only if it met all of the following criteria: an original *p*-value < 0.05, an FDR-corrected *p*-value < 0.05, and a Cohen’s *d* value > 0.2.

Then, machine learning algorithms were employed to further investigate the feasibility of constructing a depression identification method based on emotional elicitation differences and HRV features, from the perspective of classification accuracy. Prior to training, several machine learning algorithms, including Support Vector Machine (SVM) [29], K-Nearest Neighbors (KNN) [30], Decision Tree (DT), and Random Forest and Extremely Randomized Trees Classifier (ERTC) [31], were evaluated on a smaller randomly selected dataset. The results indicated that ERTC outperformed the other algorithms. Therefore, ERTC was selected for the construction of the machine learning model.

The construction of the machine learning model was implemented using the Scikit-learn algorithm package in Python [32]. The train_test_split function from the package was employed to divide the feature set into training and testing sets in a 7:3 ratio. To optimize model accuracy while mitigating overfitting risks, we employed fivefold cross-validation during model training. Bayesian optimization was subsequently applied for hyperparameter tuning of the ERTC. The receiver operating characteristic curve (ROC) was plotted to evaluate and visualize the model's effectiveness in screening IWDs. Additionally, the model's accuracy, area under the ROC (AUC) value, precision, and other metrics were calculated to assess its classification performance.

## Results

Based on the preceding discussion, this study will report and analyze results from the following two perspectives:

1. First, we present a comparative analysis of emotional elicitation in IWDs and HCs based on HRV features. This analysis employs Mann–Whitney *U* tests and examines inter-group distribution patterns to characterize emotional responses within each group and identify significant differences in elicitation efficacy between the two groups.
2. Subsequently, we employ an ERTC-based machine learning model to empirically validate key findings from the preceding HRV analysis. Leveraging these validated insights, we further develop a novel depression screening framework grounded in differential emotional responsivity.

As a reference and comparison, the subjective ratings of HCs and IWDs for the different emotional stimuli are presented in Table 4. Regarding the effectiveness of emotional induction, all stimuli successfully elicited their target emotions, as evidenced by higher scores in the corresponding emotional dimensions and lower scores in others. Furthermore, the observed arousal levels were sufficiently high. This indicates that the emotional provocation was effective in both HCs and IWDs. Concerning the differences between HCs and IWDs, several disparities in the rating patterns were observed: for the anger-eliciting stimulus, a subtle difference was noted in the sadness rating; for the fear-eliciting stimulus, a pronounced difference was found in valence; for the happiness-eliciting stimulus, a marked difference emerged in the happiness rating; and for the sadness-eliciting stimulus, evident differences were identified in the ratings of sadness, happiness, and valence.

**Table 4:** Subject scale information

Group	Emotion	Happy Score	Sad Score	Angry Score	Fear Score	Arousal	Valence
*HC*	Anger	2.01 ± 1.19	3.2 ± 1.97	5.33 ± 2.24	1.62 ± 1.4	5.87 ± 2.03	3.52 ± 1.27
	Fear	1.29 ± 0.75	2.26 ± 1.84	1.68 ± 1.55	6.81 ± 1.93	7.91 ± 1.41	2.48 ± 1.78
	Happiness	6.36 ± 1.94	1.63 ± 1.47	1.2 ± 0.89	1.21 ± 0.66	6.03 ± 1.94	6.83 ± 1.44
	Sadness	1.88 ± 1.13	6.24 ± 1.81	2.2 ± 2.04	1.52 ± 1.32	6.23 ± 1.69	3.47 ± 1.44
*IWD*	Anger	1.82 ± 1.2	2.98 ± 2.03	5.26 ± 2.18	1.83 ± 1.85	5.64 ± 2.2	3.44 ± 1.2
	Fear	1.42 ± 0.9	2.88 ± 2.57	1.68 ± 1.61	6.8 ± 1.91	7.86 ± 1.55	3.04 ± 1.88
	Happiness	6.03 ± 2.14	1.89 ± 1.61	1.29 ± 0.99	1.34 ± 1.01	6.23 ± 1.88	6.81 ± 1.52
	Sadness	1.46 ± 0.73	6.6 ± 1.76	1.95 ± 1.76	1.87 ± 1.55	6.14 ± 2.02	3.1 ± 1.55

**Fig. 2:**
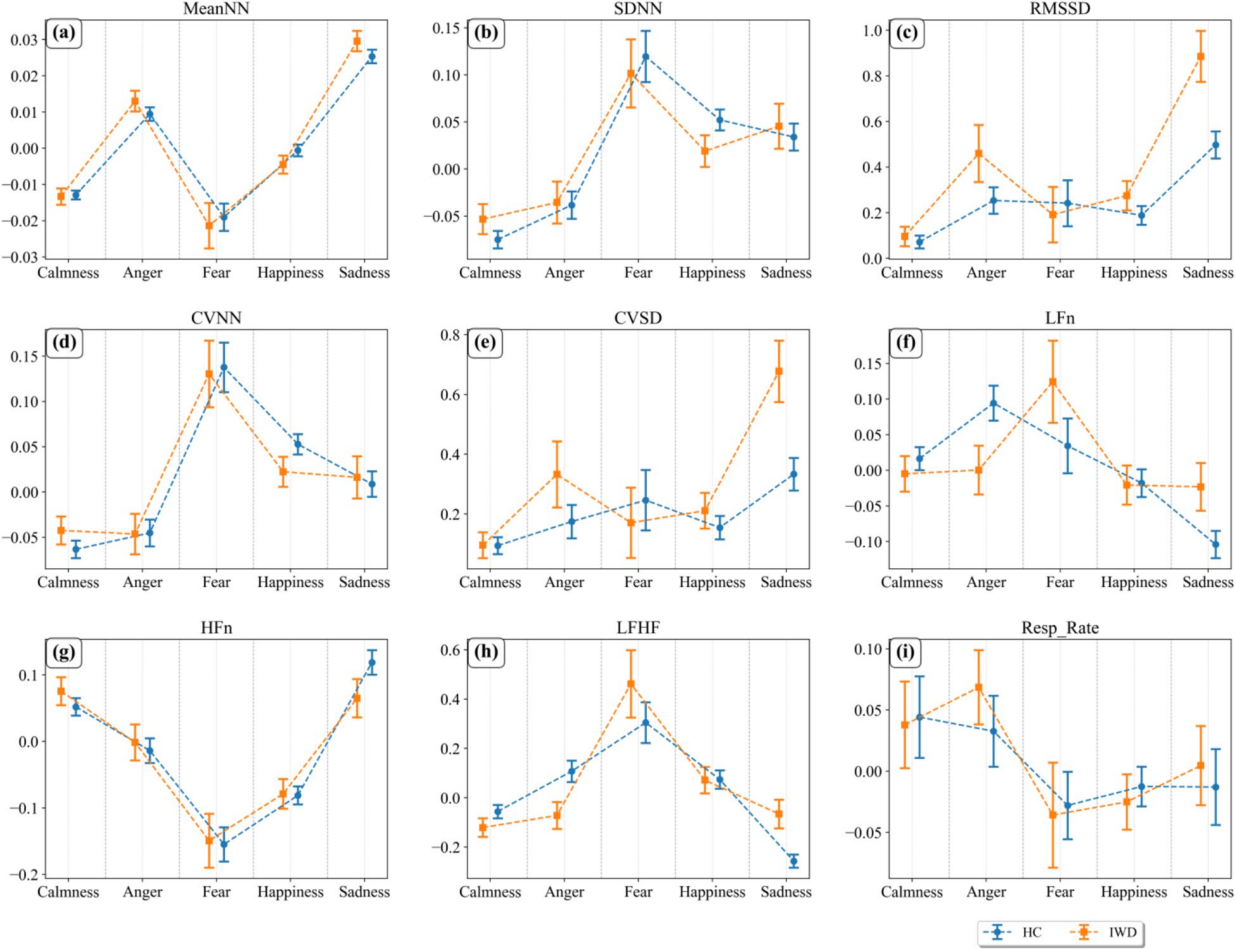
Distribution of typical features

Table 5 and Fig. 2 presented the alterations in HRV features and the corresponding statistical analyses for both HCs and IWDs across emotional conditions. The results demonstrate that HRV features exhibited substantial variation across different emotions and provided effective discriminative power for both groups, although the differentiation was more pronounced in HCs. In the between-group comparison, nearly half of the HRV features showed significant and discriminative differences under the sadness condition. In contrast, only a limited number of features displayed marked differences for the other three emotional states.

**Table 5:** Statistical information on typical features

	Calm		Angry		Fear		Happy		Sad	
	HCs	IWDs	HCs	IWDs	HCs	IWDs	HCs	IWDs	HCs	IWDs
MeanNN	-0.013±0.042	-0.013±0.051	0.009±0.045	0.013±0.046	-0.019±0.066	-0.021±0.076	-0.001±0.051	-0.005±0.052	0.025±0.048	0.030±0.047
SDNN	-0.075±0.322	-0.053±0.361	-0.038±0.351	-0.036±0.359	0.120±0.478	0.102±0.439	0.052±0.353	**0.019±0.353**	0.034±0.364	0.045±0.406
RMSSD	0.072±0.988	0.096±0.988	0.254±1.524	0.460±2.165	0.242±2.045	0.191±1.685	0.188±1.503	0.275±1.552	0.497±1.632	**0.885±2.014**
CVNN	-0.063±0.330	-0.042±0.349	-0.045±0.359	-0.046±0.360	0.138±0.482	0.130±0.446	0.053±0.358	0.022±0.347	0.009±0.359	0.016±0.395
CVSD	0.094±0.989	0.096±0.989	0.175±1.471	0.333±1.912	0.246±2.054	0.171±1.629	0.154±1.449	0.211±1.446	0.333±1.496	**0.677±1.852**
LFn	0.016±0.561	-0.005±0.562	0.094±0.592	**0.000±0.553**	0.034±0.677	**0.124±0.700**	-0.018±0.617	-0.021±0.574	-0.104±0.482	**-0.023±0.569**
HFn	0.052±0.453	0.076±0.472	-0.014±0.446	-0.002±0.437	-0.155±0.454	-0.149±0.490	-0.081±0.430	-0.079±0.470	0.119±0.465	0.065±0.489
LFHF	-0.056±0.932	-0.121±0.845	0.107±1.049	**-0.072±0.881**	0.305±1.457	0.462±1.656	0.074±1.179	0.071±1.125	-0.257±0.675	**-0.066±0.986**
Resp_rate	0.044±0.273	0.038±0.191	0.033±0.238	0.069±0.164	-0.028±0.222	-0.036±0.232	-0.012±0.228	-0.025±0.208	-0.013±0.250	0.005±0.174

a) Underlined values denote a significant within-group change (*p* < 0.05 & p-fdr < 0.05 and effect size > 0.2) in the HRV feature between the baseline (calm) state and the specified emotional state for that group.

b) Boldface values indicate a significant between-group difference (*p* < 0.05 and p-fdr < 0.05 and effect size > 0.2) in the HRV feature between individuals with depression (IWDs) and healthy controls (HCs) under the specified emotional state

Comprehensive details are provided as follows:

1. Regarding respiration rate’s reflection of emotional elicitation, it was found to be effective in indicating the emotional state; however, no significant differences were observed between IWDs and HCs. Consequently, the influence of respiration rate on HRV manifestations of differences in emotional elicitation between the two groups can be largely ruled out.
2. For the angry emotion: the direction of emotional elicitation reflected by MeanNN, RMSSD, and CVSD was consistent between IWDs and HCs, but more pronounced in IWDs. However, these specific features did not reach statistical significance. Conversely, LFn was less effective in reflecting the angry elicitation in IWDs; thus, differences were also noted for LFn and LFHF.
3. For the fear emotion: differences primarily manifested in LFn and consequently in LFHF, but LFHF did not reach statistical significance
4. For the happy emotion: the direction of emotional elicitation reflected by SDNN, RMSSD, and CVNN was consistent between IWDs and HCs, but less pronounced in IWDs. However, only SDNN reached statistical significance.
5. For the sad emotion: the direction of emotional elicitation reflected by the time-domain features (MeanNN, RMSSD, CVSD) was consistent between IWDs and HCs, but more pronounced in IWDs. In contrast, the frequency-domain features (LFn, HFn, LFHF) were less effective in reflecting sad elicitation in IWDs, while they effectively reflected it in HCs. Among these, RMSSD, CVSD, LFn, and LFHF demonstrated statistically significant differences.

These findings demonstrate that HRV features are effective indicators of emotional elicitation in both IWDs and HCs groups. However, intergroup differences persist in either the direction or magnitude of elicited responses: the sadness-elicited response was more pronounced in IWD than in HC, Whereas the happiness-elicited response was less pronounced in IWD. Furthermore, differences in angry and fear elicitation primarily manifested in the frequency-domain features of HRV. But the differences under the other three emotions are far less pronounced than under sadness.

**Fig. 3:**
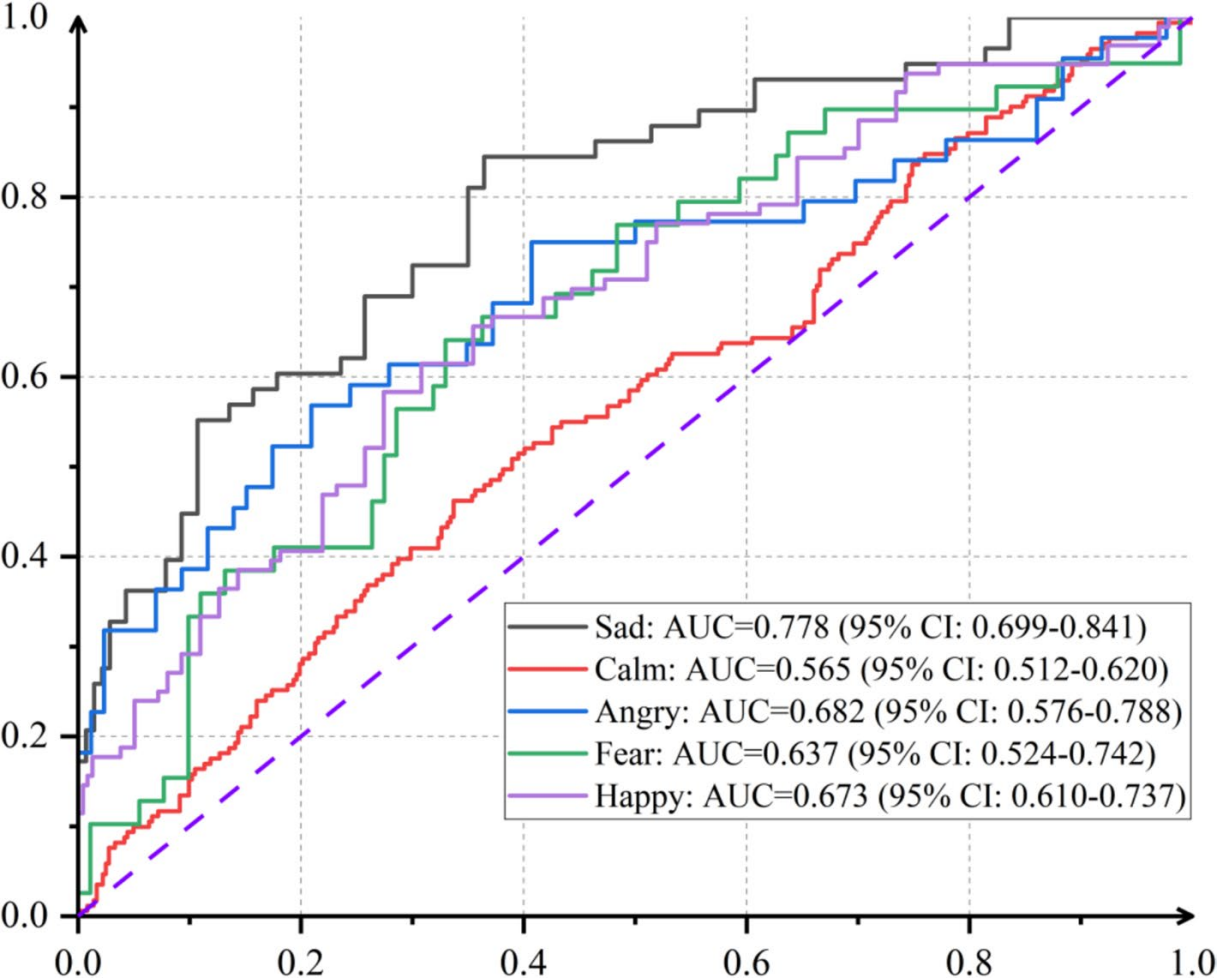
ROCs of ERTC in screening depression under different emotional stimulis

Based on the observed elicitation differences, emotion-specific ERTC models were constructed for each affective state. For comparative purposes, a calm-state model was established using the raw features without emotional induction. Additionally, logistic regression was employed as a control model against the ERTC. The results are presented in Table 6 and Fig. 3. Classification accuracy, AUC values, and ROC curves consistently demonstrated that:

1. The sadness-elicited model most effectively discriminated between IWD and HC groups (accuracy = 76.8%, 95% CI 0.707–0.823; AUC = 0.778, 95% CI 0.699–0.841)
2. Models for anger (accuracy = 71.5%, 95% CI 0.638–0.792; AUC = 0.682, 95% CI 0.576–0.788) and happiness (accuracy = 73.0%, 95% CI 0.682–0.775; AUC = 0.673, 95% CI 0.610–0.737) showed intermediate performance.
3. The fear-elicited model achieved slightly lower accuracy (accuracy = 68.5%, 95% CI 0.600–0.762; AUC = 0.637, 95% CI 0.524–0.742).
4. The calm-state model yielded the lowest accuracy (accuracy = 67.9%, 95% CI 0.642–0.719; AUC = 0.565, 95% CI 0.512–0.62), serving as the performance baseline.

**Table 6:** Accuracy results in screening depression under different emotional stimulis

*Emotion*	ML method	Accuracy (95% CI)	AUC (95% CI)	Precision (95% CI)
Sad	ERTC	0.768 (0.707, 0.823)	0.778 (0.699, 0.841)	0.8 (0.615, 0.95)
	LR	0.727 (0.661, 0.788)	0.67 (0.577, 0.75)	0.75 (0.583, 0.918)
Happy	ERTC	0.73 (0.682, 0.775)	0.673 (0.61, 0.737)	0.6 (0.417, 0.79)
	LR	0.709 (0.658, 0.76)	0.632 (0.559, 0.695)	0.667 (0.512, 0.823)
Anrgy	ERTC	0.715 (0.638, 0.792)	0.682 (0.576, 0.788)	0.818 (0.648, 0.938)
	LR	0.697 (0.633, 0.761)	0.584 (0.487, 0.674)	0.523 (0.385, 0.672)
Fear	ERTC	0.685 (0.6, 0.762)	0.637 (0.524, 0.742)	0.562 (0.415, 0.703)
	LR	0.631 (0.554, 0.708)	0.574 (0.475, 0.673)	0.714 (0.586, 0.853)
Calm	ERTC	0.679 (0.642, 0.719)	0.565 (0.512, 0.62)	0.633 (0.508, 0.762)
	LR	0.679 (0.64, 0.715)	0.511 (0.455, 0.563)	0.659 (0.534, 0.786)

The findings suggest that HRV features under emotional induction provide a more effective basis for differentiating IWDs from HCs than calm-state features, with sadness evidencing the greatest discriminative validity. From an HRV standpoint, the study thereby bolsters existing evidence for a heightened sensitivity to sad stimuli in IWDs, reflected in an augmented physiological response. Nevertheless, the anticipated attenuation of the emotional response to happiness in IWDs was not clearly demonstrated. This absence of a pronounced effect may be attributed to the emotional provocation paradigm; the subjective ratings revealed that while the reported intensity of emotions differed between groups, the absence of concurrent significant differences in valence and arousal levels likely contributed to the diminished group contrast.

## Discussion

This study systematically compared emotion-elicited HRV features between IWDs and HCs, revealing population-specific differential responses across affective states. Through this approach, the research not only validates existing literature but also proposes an affective computing-based optimization framework for depression screening. Furthermore, it advances the mechanistic understanding of depression-related physiological responses under varied emotional conditions.

Both IWDs and HCs underwent identical emotion induction protocols across four basic emotions (sad, happy, fear, angry) and a resting-state condition (calm), with synchronized collection of self-report scales and physiological data; comparative analysis of HRV feature elicitation revealed broadly consistent directional patterns and response magnitudes between groups for most features, although critical differences emerged wherein Sad elicitation manifested heightened responsiveness in IWDs while Happy elicitation demonstrated attenuated responsiveness in this group. These results are consistent with existing psychological research [7, 8] and the emotional scale data obtained in the experiment.

Building upon the identified intergroup differences under emotional conditions, this study constructed five emotion-specific depression screening models. The sadness-based model achieved near-optimal performance (accuracy ≈ 77%), while models utilizing anger or happiness elicitation attained moderate accuracy (≈ 70%). In contrast, the resting-state model demonstrated suboptimal discriminative capability. These results collectively validate the efficacy of affective computing approaches in depression identification. Comparative analysis with existing literature (Table 7) reveals that our sadness-elicitation model remains highly competitive despite methodological variations. Notwithstanding accuracy discrepancies relative to certain studies—attributable to divergent experimental materials, participant profiles, and our single-emotion induction paradigm—the present findings retain substantive reference value for developing targeted diagnostic frameworks. For instance, the depression testing scheme used by Kim [19], such as the MAC test, could be further optimized by focusing more on sadness and happiness while reducing the emphasis on other emotional states. This approach could potentially shorten the duration of the testing scheme and improve its efficiency. Alternatively, a promising future direction would be to consolidate a resting state assessment with a brief sadness induction, which could streamline the entire testing protocol to within 5–10 min.

**Table 7:** Comparisons with other studies

Authors	Subjects	Classification	Signal/features	Affective computing or not	Accuracy
Byun (2019) [33]	MDD:37; HC:41	SVM	HRV	Not	74%
Geng (2023) [34]	MDD:40; HC:40	BO-ERTC	HRV	Not	86%
Kuang (2017) [35]	All female; MDD:38; HC:38	Bayesian Networks	HRV	Not	71%-86%
Sun (2016) [36]	MDD:44; HC:47	Logistic Regression	HRV	Not	79%
Lyu (2024) [16]	BPD:77; MDD:53; HC:79	SVM	EDA	Yes	78%
Lan (2023) [17]	MDD:33; HC:40	CNN	EEG	Yes	AUC > 84%
Kim (2019) [19]	MDD:10; HC:14	Neural Fuzzy Algorithm	HRV	Yes	86%
This study	IWD:30; HC:66	ETRC	HRV	Yes	76.80%

Based on the findings of this study, several promising research directions can be proposed. First, future investigations could explore the integration of additional physiological signals, such as EEG and EDA, to comprehensively analyze the physiological mechanisms from multiple perspectives and provide a more robust theoretical foundation for depression screening. Second, further efforts may focus on streamlining the methodology, for instance, by utilizing more accessible PPG signals or exclusively selecting features that are independent of segment duration, thereby developing screening approaches that are both efficient and rapid while maintaining diagnostic validity.

Despite its contributions, this study has several limitations that point to avenues for future research. First, the reliability of frequency-domain HRV indices derived from 1-min RR intervals is suboptimal, although it is sufficient for use [26, 27]. Consequently, future studies should utilize segments of at least 90 s. Second, the lack of a pronounced difference between IWDs and HCs in response to happiness may be attributed to the efficacy of the emotional induction. The subjective ratings showed a difference in intensity but not in valence or arousal, potentially obscuring group differences. Future work should employ a wider array of happiness-eliciting stimuli to elucidate the underlying mechanisms. Third, the feature profile under anger was somewhat anomalous, with group differences being slightly more marked than anticipated. This may be due to the stimulus material, which potentially co-elicited sadness, as suggested by the rating scores, despite the overall successful induction of anger. A review of the video content confirms this potential for mixed emotions. Finally, as an initial exploration, this study has scope for significant refinement in areas such as stimulus selection and framework development. Future iterations should also consider expanding the participant pool to include a wider age range and more diverse socio-demographic backgrounds.

## Conclusion

Leveraging discrete emotion induction protocols, this study systematically analyzed HRV-mediated elicitation patterns across affective states, revealing clinically significant differential responsiveness between IWDs and HCs that empirically substantiates heightened sadness sensitivity in IWDs. These findings provide empirical guidance for optimizing affective computing-based depression screening algorithms while advancing the mechanistic understanding of disorder-specific physiological responses under varied emotional conditions.

## Supplementary Information

Supplementary Material 1. Figure S1. Typical ECG signal processing. Table S1. Statistical information of HCs. Table S2. Statistical information of IWDs. Table S3. Statistical information between HCs and IWDs. Figure S2. ERTC for Calmness. Figure S3. ERTC for Anger. Figure S4. ERTC for Fear. Figure S5. ERTC for Happiness. Figure S6. ERTC for Sadness. Figure S7. Logistic Regression for Calmness. Figure S8. Logistic Regression for Anger. Figure S9. Logistic Regression for Fear. Figure S10. Logistic Regression for Happiness. Figure S11. Logistic Regression for Sadness.

## Data Availability

The datasets used and/or analysed during the current study are available from the corresponding author on reasonable request.

## Abbreviation

IWD: Individual with depression
HC: Healthy control
HRV: Heart rate variability
ECG: Electrocardiogram
ERTC: Extremely Randomized Trees Classifier
ROC: Receiver operating characteristic curve
AUC: Area under the ROC
