# Supplementary material for: A heart rate variability-driven framework for depression screening leveraging emotion-elicited autonomic divergence
Source: J Physiol Anthropol. 2025 Dec 24;44:33. doi: 10.1186/s40101-025-00414-6 (PMC12729146; doi:10.1186/s40101-025-00414-6)
Supplement: Supplementary file 1 — Supplementary Material 1. Figure S1. Typical ECG signal processing. Table S1. Statistical information of HCs. Table S2. Statistical information of IWDs. Table S3. Statistical information between HCs and IWDs. Figure S2. ERTC for Calmness. Figure S3. ERTC for Anger. Figure S4. ERTC for Fear. Figure S5. ERTC for Happiness. Figure S6. ERTC for Sadness. Figure S7. Logistic Regression for Calmness. Figure S8. Logistic Regression for Anger. Figure S9. Logistic Regression for Fear. Figure S10. Logistic Regression for Happiness. Figure S11. Logistic Regression for Sadness. [file 40101_2025_414_MOESM1_ESM.docx]

Supplementary Material

# Typical ECG signal processing situation

#
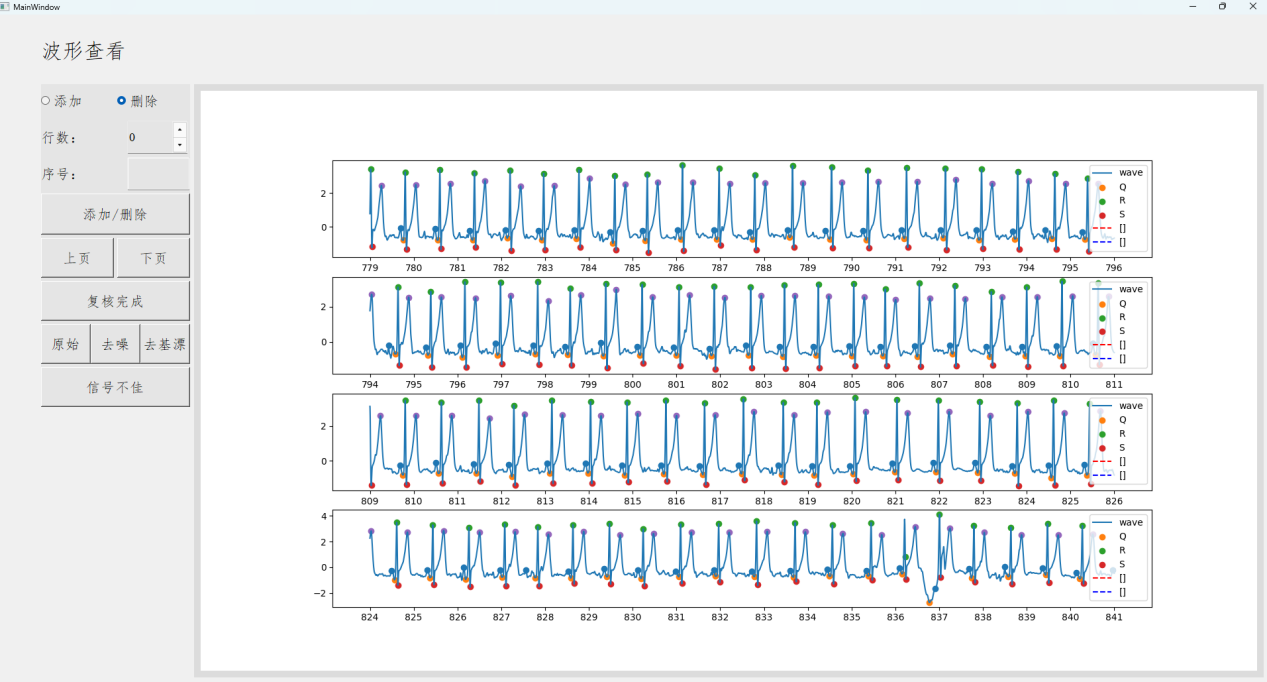


**Supplementary Figure 1.** Typical ECG signal processing

# P-Value & P-FDR & Cohen's d effect size

## Statistical information of HCs

| Feature | Type | Calm-Angry | Calm-Fear | Calm-Happy | Calm-Sad | Angry-Fear | Angry-Happy | Angry-Sad | Fear-Happy | Fear-Sad | Happy-Sad |
| --- | --- | --- | --- | --- | --- | --- | --- | --- | --- | --- | --- |
| MeanNN | Cohen's d | -0.521 | 0.128 | -0.266 | -0.869 | 0.534 | 0.205 | -0.342 | -0.335 | -0.815 | -0.520 |
| SDNN | Cohen's d | -0.111 | -0.541 | -0.378 | -0.323 | -0.395 | -0.257 | -0.202 | 0.175 | 0.211 | 0.051 |
| RMSSD | Cohen's d | -0.151 | -0.128 | -0.091 | -0.335 | 0.007 | 0.043 | -0.154 | 0.033 | -0.143 | -0.199 |
| CVNN | Cohen's d | -0.053 | -0.549 | -0.338 | -0.212 | -0.450 | -0.273 | -0.150 | 0.217 | 0.320 | 0.122 |
| CVSD | Cohen's d | -0.068 | -0.114 | -0.048 | -0.198 | -0.042 | 0.014 | -0.107 | 0.057 | -0.051 | -0.122 |
| LFn | Cohen's d | -0.136 | -0.031 | 0.058 | 0.225 | 0.096 | 0.185 | 0.370 | 0.083 | 0.251 | 0.152 |
| HFn | Cohen's d | 0.146 | 0.456 | 0.301 | -0.146 | 0.314 | 0.154 | -0.291 | -0.169 | -0.592 | -0.450 |
| LFHF | Cohen's d | -0.168 | -0.339 | -0.123 | 0.236 | -0.163 | 0.030 | 0.417 | 0.185 | 0.562 | 0.326 |
| Resp_Rate | Cohen's d | 0.045 | 0.290 | 0.236 | 0.218 | 0.264 | 0.196 | 0.187 | -0.068 | -0.064 | 0.002 |
| MeanNN | p_fdr | 0.000 | 0.895 | 0.000 | 0.000 | 0.000 | 0.000 | 0.000 | 0.000 | 0.000 | 0.000 |
| SDNN | p_fdr | 0.123 | 0.000 | 0.000 | 0.000 | 0.000 | 0.000 | 0.000 | 0.157 | 0.037 | 0.228 |
| RMSSD | p_fdr | 0.049 | 0.762 | 0.795 | 0.000 | 0.571 | 0.220 | 0.048 | 0.846 | 0.019 | 0.000 |
| CVNN | p_fdr | 0.689 | 0.000 | 0.000 | 0.000 | 0.000 | 0.000 | 0.004 | 0.030 | 0.000 | 0.016 |
| CVSD | p_fdr | 0.725 | 0.725 | 0.725 | 0.055 | 0.830 | 0.725 | 0.271 | 0.725 | 0.518 | 0.055 |
| LFn | p_fdr | 0.029 | 0.597 | 0.032 | 0.000 | 0.037 | 0.000 | 0.000 | 0.324 | 0.032 | 0.112 |
| HFn | p_fdr | 0.003 | 0.000 | 0.000 | 0.002 | 0.000 | 0.004 | 0.000 | 0.004 | 0.000 | 0.000 |
| LFHF | p_fdr | 0.001 | 0.000 | 0.032 | 0.000 | 0.198 | 0.159 | 0.000 | 0.017 | 0.000 | 0.000 |
| Resp_Rate | p_fdr | 0.908 | 0.518 | 0.518 | 0.518 | 0.518 | 0.518 | 0.617 | 0.647 | 0.707 | 0.908 |
| MeanNN | p_value | 0.000 | 0.895 | 0.000 | 0.000 | 0.000 | 0.000 | 0.000 | 0.000 | 0.000 | 0.000 |
| SDNN | p_value | 0.099 | 0.000 | 0.000 | 0.000 | 0.000 | 0.000 | 0.000 | 0.141 | 0.026 | 0.228 |
| RMSSD | p_value | 0.025 | 0.609 | 0.715 | 0.000 | 0.400 | 0.132 | 0.019 | 0.846 | 0.006 | 0.000 |
| CVNN | p_value | 0.689 | 0.000 | 0.000 | 0.000 | 0.000 | 0.000 | 0.003 | 0.027 | 0.000 | 0.013 |
| CVSD | p_value | 0.543 | 0.618 | 0.652 | 0.011 | 0.830 | 0.517 | 0.081 | 0.511 | 0.207 | 0.009 |
| LFn | p_value | 0.012 | 0.597 | 0.017 | 0.000 | 0.026 | 0.000 | 0.000 | 0.292 | 0.019 | 0.090 |
| HFn | p_value | 0.002 | 0.000 | 0.000 | 0.002 | 0.000 | 0.004 | 0.000 | 0.004 | 0.000 | 0.000 |
| LFHF | p_value | 0.001 | 0.000 | 0.026 | 0.000 | 0.198 | 0.143 | 0.000 | 0.012 | 0.000 | 0.000 |
| Resp_Rate | p_value | 0.843 | 0.093 | 0.182 | 0.259 | 0.107 | 0.248 | 0.370 | 0.453 | 0.566 | 0.908 |

**Supplementary Table 1.** Statistical information of HCs

## Statistical information of IWDs

| Feature | Type | Calm-Angry | Calm-Fear | Calm-Happy | Calm-Sad | Angry-Fear | Angry-Happy | Angry-Sad | Fear-Happy | Fear-Sad | Happy-Sad |
| --- | --- | --- | --- | --- | --- | --- | --- | --- | --- | --- | --- |
| MeanNN | Cohen's d | -0.535 | 0.140 | -0.172 | -0.866 | 0.587 | 0.352 | -0.354 | -0.287 | -0.869 | -0.679 |
| SDNN | Cohen's d | -0.049 | -0.408 | -0.202 | -0.262 | -0.352 | -0.154 | -0.211 | 0.219 | 0.135 | -0.071 |
| RMSSD | Cohen's d | -0.239 | -0.079 | -0.136 | -0.539 | 0.135 | 0.104 | -0.204 | -0.053 | -0.365 | -0.353 |
| CVNN | Cohen's d | 0.011 | -0.463 | -0.186 | -0.160 | -0.449 | -0.195 | -0.165 | 0.289 | 0.277 | 0.017 |
| CVSD | Cohen's d | -0.170 | -0.063 | -0.092 | -0.422 | 0.090 | 0.075 | -0.183 | -0.027 | -0.286 | -0.291 |
| LFn | Cohen's d | -0.009 | -0.217 | 0.028 | 0.032 | -0.204 | 0.037 | 0.042 | 0.239 | 0.240 | 0.004 |
| HFn | Cohen's d | 0.167 | 0.472 | 0.328 | 0.022 | 0.323 | 0.169 | -0.143 | -0.148 | -0.437 | -0.301 |
| LFHF | Cohen's d | -0.057 | -0.539 | -0.195 | -0.061 | -0.438 | -0.138 | -0.007 | 0.306 | 0.421 | 0.128 |
| Resp_Rate | Cohen's d | -0.173 | 0.348 | 0.309 | 0.182 | 0.521 | 0.473 | 0.379 | -0.050 | -0.198 | -0.149 |
| MeanNN | p_fdr | 0.000 | 0.738 | 0.004 | 0.000 | 0.000 | 0.000 | 0.000 | 0.176 | 0.000 | 0.000 |
| SDNN | p_fdr | 0.417 | 0.000 | 0.001 | 0.001 | 0.010 | 0.070 | 0.064 | 0.112 | 0.215 | 0.750 |
| RMSSD | p_fdr | 0.214 | 0.744 | 0.421 | 0.000 | 0.567 | 0.654 | 0.001 | 0.714 | 0.000 | 0.000 |
| CVNN | p_fdr | 0.921 | 0.000 | 0.002 | 0.085 | 0.001 | 0.015 | 0.130 | 0.028 | 0.015 | 0.375 |
| CVSD | p_fdr | 0.471 | 0.766 | 0.718 | 0.000 | 0.766 | 0.766 | 0.005 | 0.766 | 0.005 | 0.000 |
| LFn | p_fdr | 0.898 | 0.317 | 0.856 | 0.856 | 0.429 | 0.856 | 0.856 | 0.317 | 0.317 | 0.898 |
| HFn | p_fdr | 0.057 | 0.000 | 0.000 | 0.867 | 0.001 | 0.031 | 0.112 | 0.076 | 0.000 | 0.000 |
| LFHF | p_fdr | 0.418 | 0.003 | 0.033 | 0.919 | 0.025 | 0.337 | 0.481 | 0.090 | 0.005 | 0.090 |
| Resp_Rate | p_fdr | 0.712 | 0.675 | 0.441 | 0.687 | 0.441 | 0.441 | 0.675 | 0.987 | 0.687 | 0.687 |
| MeanNN | p_value | 0.000 | 0.738 | 0.003 | 0.000 | 0.000 | 0.000 | 0.000 | 0.158 | 0.000 | 0.000 |
| SDNN | p_value | 0.376 | 0.000 | 0.000 | 0.000 | 0.004 | 0.042 | 0.032 | 0.078 | 0.172 | 0.750 |
| RMSSD | p_value | 0.107 | 0.744 | 0.252 | 0.000 | 0.397 | 0.523 | 0.000 | 0.642 | 0.000 | 0.000 |
| CVNN | p_value | 0.921 | 0.000 | 0.001 | 0.060 | 0.000 | 0.007 | 0.104 | 0.017 | 0.006 | 0.338 |
| CVSD | p_value | 0.236 | 0.766 | 0.431 | 0.000 | 0.572 | 0.673 | 0.002 | 0.744 | 0.002 | 0.000 |
| LFn | p_value | 0.876 | 0.095 | 0.545 | 0.684 | 0.172 | 0.509 | 0.642 | 0.053 | 0.065 | 0.898 |
| HFn | p_value | 0.040 | 0.000 | 0.000 | 0.867 | 0.001 | 0.019 | 0.101 | 0.061 | 0.000 | 0.000 |
| LFHF | p_value | 0.335 | 0.000 | 0.013 | 0.919 | 0.008 | 0.236 | 0.433 | 0.045 | 0.001 | 0.054 |
| Resp_Rate | p_value | 0.640 | 0.338 | 0.132 | 0.486 | 0.103 | 0.051 | 0.291 | 0.987 | 0.550 | 0.416 |

**Supplementary Table 2.** Statistical information of IWDs

## Statistical information between HCs and IWDs

| Feature | Type | Calm | Angry | Fear | Happy | Sad |
| --- | --- | --- | --- | --- | --- | --- |
| MeanNN | Cohen's d | -0.0096 | 0.0781 | -0.0338 | -0.0764 | 0.0886 |
| SDNN | Cohen's d | 0.0651 | 0.0077 | -0.0385 | -0.2437 | 0.0305 |
| RMSSD | Cohen's d | 0.0247 | 0.1183 | -0.0260 | 0.0569 | 0.3010 |
| CVNN | Cohen's d | 0.0622 | -0.0033 | -0.0154 | -0.2155 | 0.0199 |
| CVSD | Cohen's d | 0.0015 | 0.0977 | -0.0391 | 0.0390 | 0.3438 |
| LFn | Cohen's d | -0.0380 | -0.2522 | 0.2216 | -0.0044 | 0.2086 |
| HFn | Cohen's d | 0.0515 | 0.0279 | 0.0118 | 0.0052 | -0.1140 |
| LFHF | Cohen's d | -0.0717 | -0.2894 | 0.1031 | -0.0022 | 0.2834 |
| Resp_Rate | Cohen's d | -0.0251 | 0.1641 | -0.0349 | -0.0568 | 0.0764 |
| MeanNN | p_fdr | 0.2350 | 0.2350 | 0.2350 | 0.2350 | 0.2350 |
| SDNN | p_fdr | 0.2005 | 0.2329 | 0.2005 | 0.0386 | 0.2329 |
| RMSSD | p_fdr | 0.2059 | 0.2034 | 0.2034 | 0.1037 | 0.0011 |
| CVNN | p_fdr | 0.2122 | 0.2122 | 0.2122 | 0.0882 | 0.2122 |
| CVSD | p_fdr | 0.2000 | 0.1675 | 0.1675 | 0.1362 | 0.0023 |
| LFn | p_fdr | 0.1097 | 0.0389 | 0.0396 | 0.1097 | 0.0396 |
| HFn | p_fdr | 0.1563 | 0.1563 | 0.1563 | 0.1563 | 0.1214 |
| LFHF | p_fdr | 0.0756 | 0.0218 | 0.1486 | 0.1642 | 0.0494 |
| Resp_Rate | p_fdr | 0.1629 | 0.1629 | 0.1629 | 0.1629 | 0.1629 |
| MeanNN | p_value | 0.2332 | 0.2270 | 0.1242 | 0.0542 | 0.2350 |
| SDNN | p_value | 0.1203 | 0.1928 | 0.1172 | 0.0077 | 0.2329 |
| RMSSD | p_value | 0.2059 | 0.1626 | 0.1627 | 0.0415 | 0.0002 |
| CVNN | p_value | 0.0936 | 0.2036 | 0.1634 | 0.0176 | 0.2122 |
| CVSD | p_value | 0.2000 | 0.1270 | 0.1340 | 0.0545 | 0.0005 |
| LFn | p_value | 0.1097 | 0.0078 | 0.0238 | 0.1094 | 0.0185 |
| HFn | p_value | 0.0968 | 0.1543 | 0.1557 | 0.1563 | 0.0243 |
| LFHF | p_value | 0.0454 | 0.0044 | 0.1188 | 0.1642 | 0.0198 |
| Resp_Rate | p_value | 0.1542 | 0.0749 | 0.1083 | 0.1629 | 0.0849 |

**Supplementary Table 3.** Statistical information between HCs and IWDs

# ROC Curves


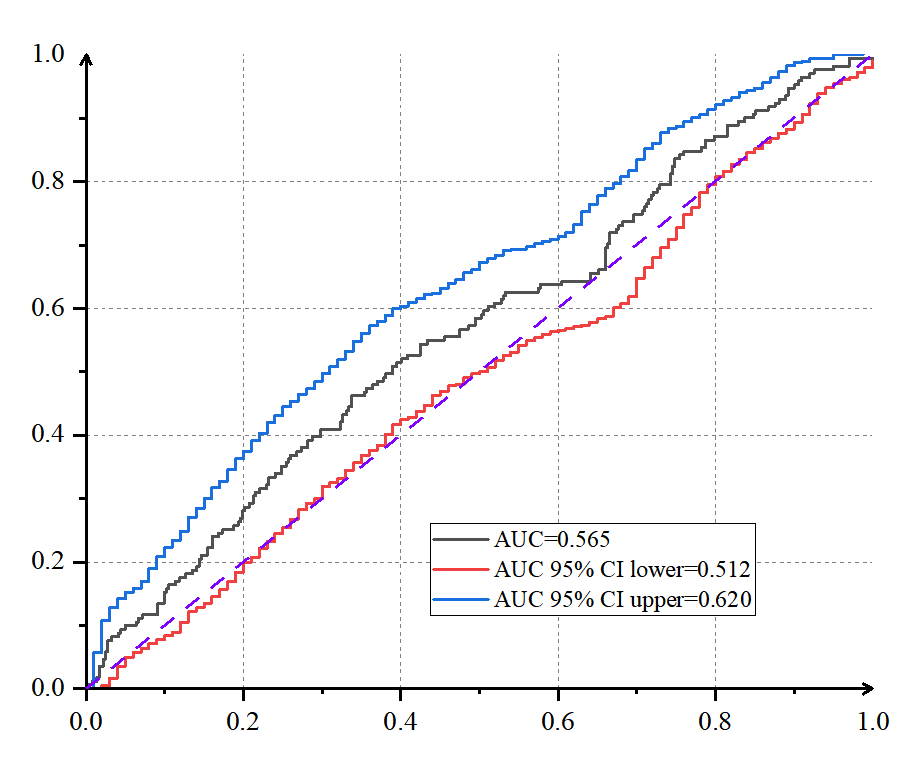


**Supplementary Figure 2.** ERTC for Calmness


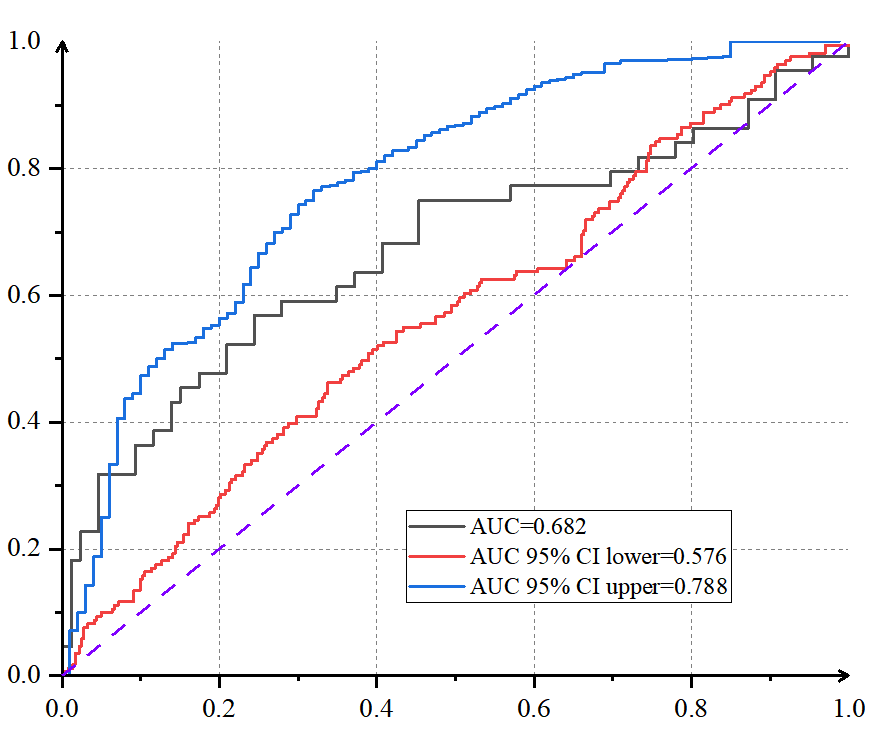


**Supplementary Figure 3.** ERTC for Anger


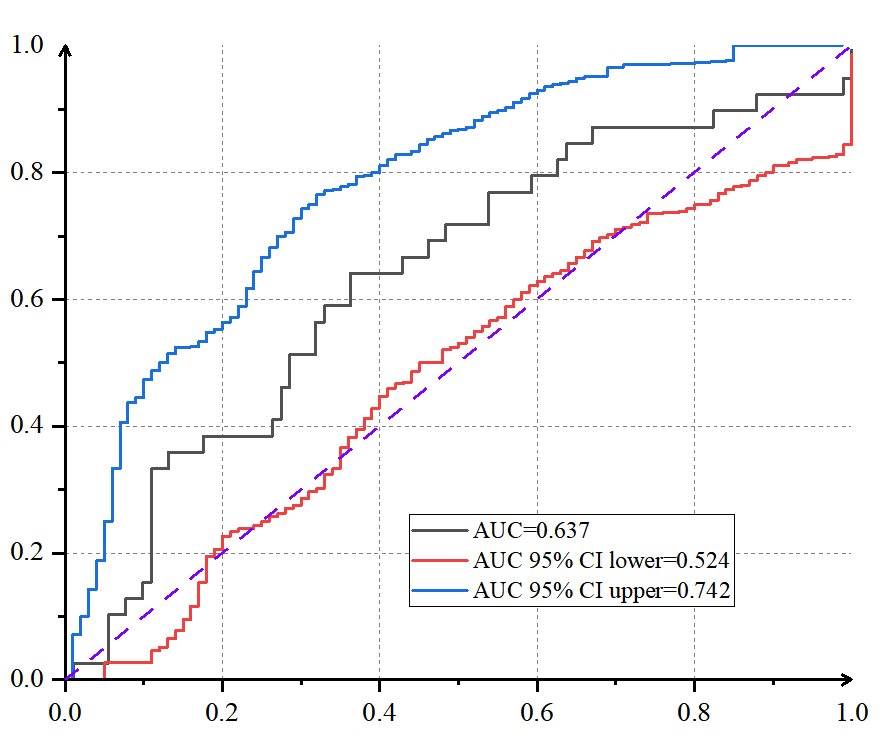


**Supplementary Figure 4.** ERTC for Fear


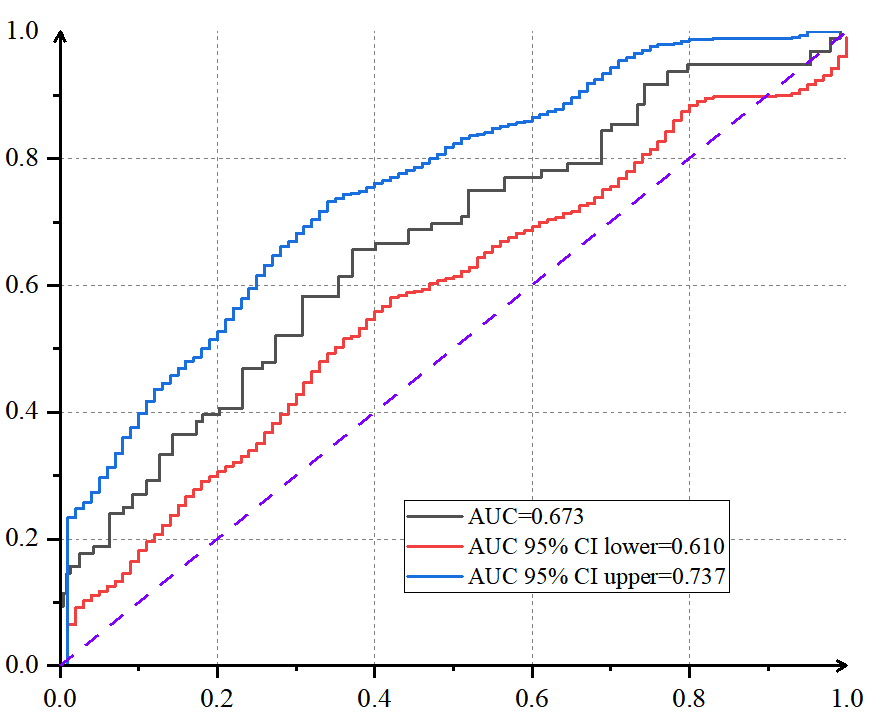


**Supplementary Figure 5.** ERTC for Happiness


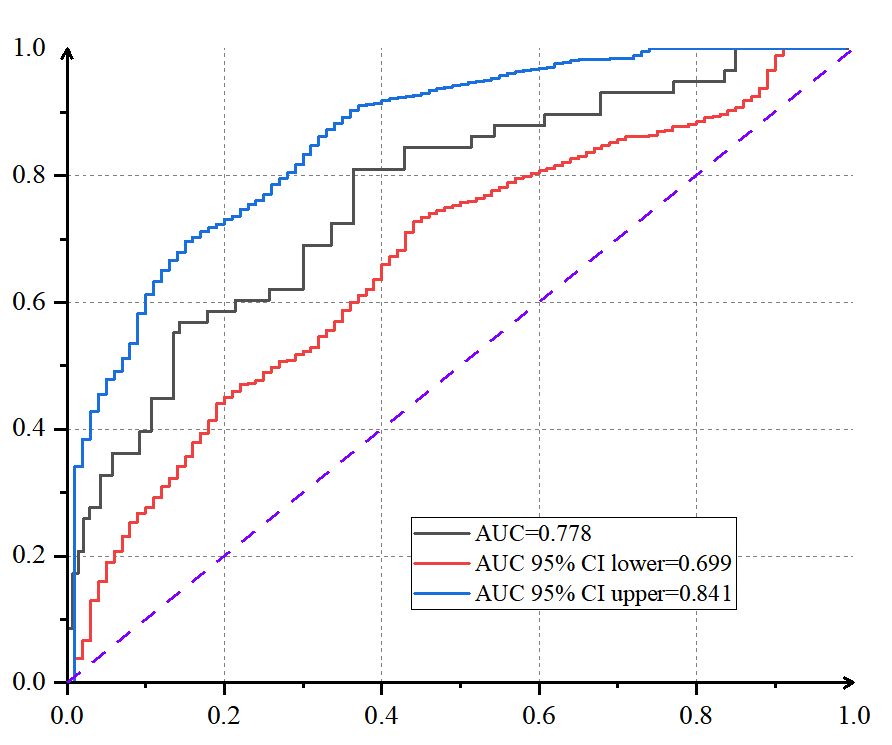


**Supplementary Figure 6.** ERTC for Sadness


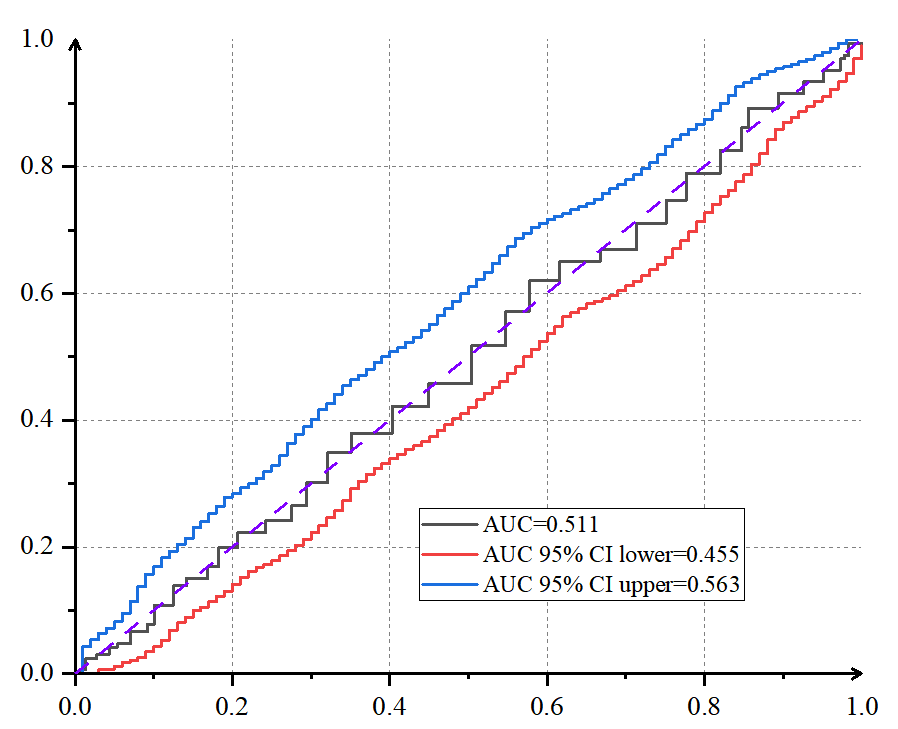


**Supplementary Figure 7.** Logistic Regression for Calmness


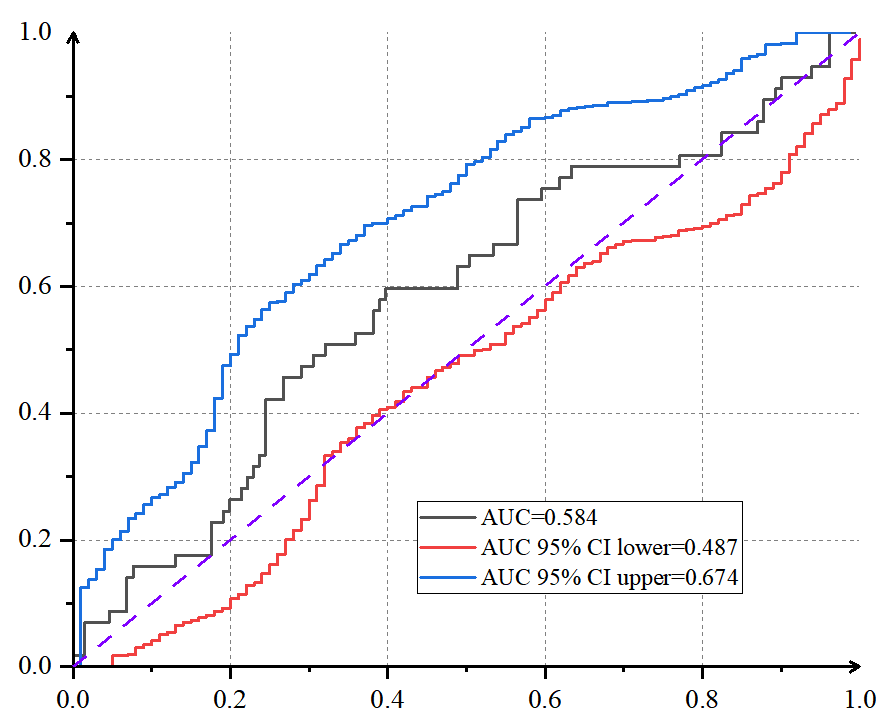


**Supplementary Figure 8.** Logistic Regression for Anger


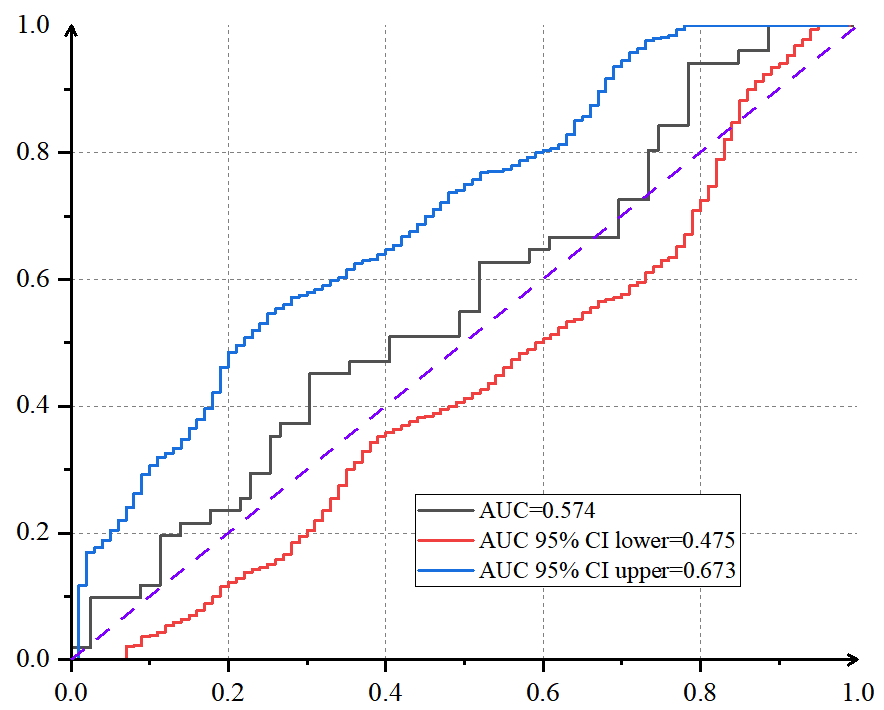


**Supplementary Figure 9.** Logistic Regression for Fear


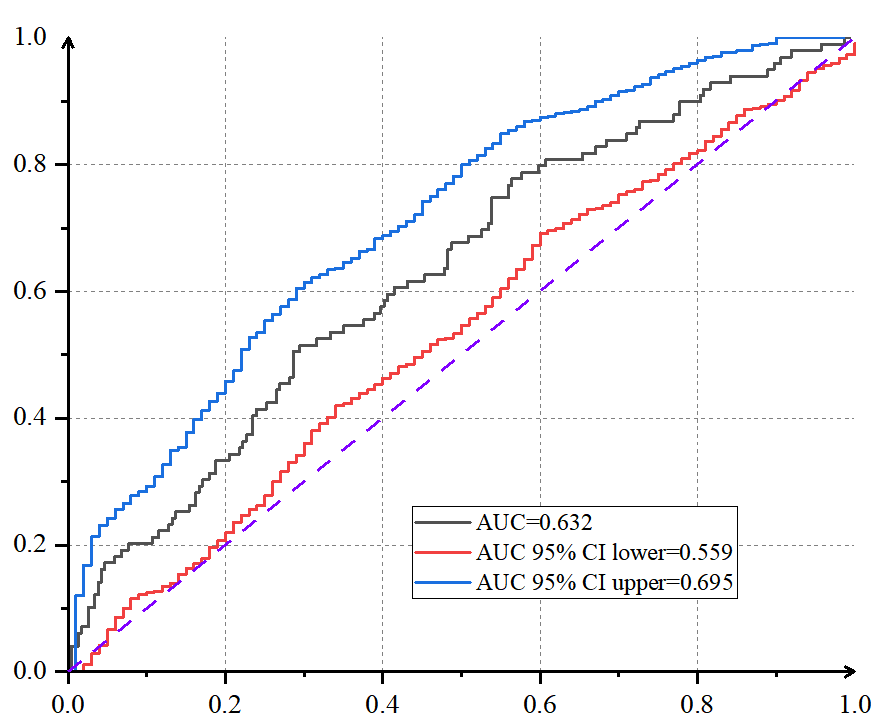


**Supplementary Figure 10.** Logistic Regression for Happiness


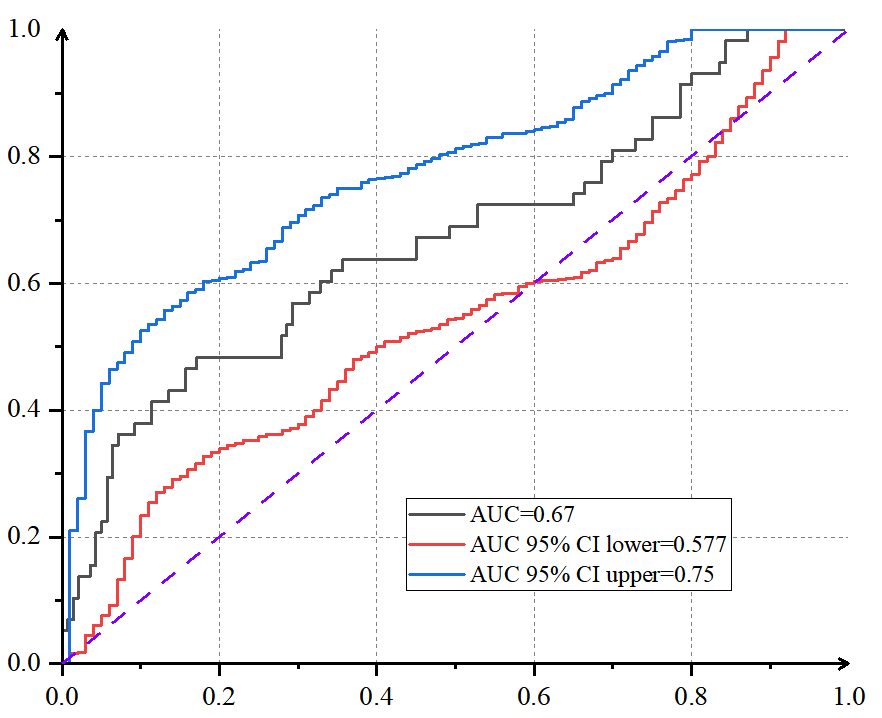


**Supplementary Figure 11.** Logistic Regression for Sadness
